# Effect of Queue Management System on Patient Satisfaction in Emergency Department; a Randomized Controlled Trial 

**DOI:** 10.22037/aaem.v9i1.1335

**Published:** 2021-09-05

**Authors:** Ali Bidari, Shabahang Jafarnejad, Nazanin Alaei Faradonbeh

**Affiliations:** 1 Emergency Medicine Department, Iran University of Medical Sciences, Tehran, Iran.

**Keywords:** Emergency service, hospital, Patient satisfaction, Waiting rooms, Pediatric emergency medicine

## Abstract

**Introduction:**

Patients’ experience in hospitals affects their satisfaction. The purpose of the present study was to assess the effect of applying a queue management system on patient satisfaction in emergency department waiting rooms.

**Methods:**

The present prospective randomized single-blinded interventional study was performed from July to August 2020 and involved 236 patients that were divided into one intervention group and one control group, each consisting of 118 patients. The mentioned patients’ perception of the waiting time and satisfaction before being visited by an emergency medicine doctor was evaluated with and without applying the queue management system.

**Results:**

The mean actual waiting time (15.5 ± 7.5 minutes) as well as the mean perceived waiting time (11.9 ± 7.4 minutes) for the intervention group were significantly lower than those of the control group with the values of 27.03 ± 8.5 and 32.8 ± 8.7 minutes, respectively (p < 0.001). The mean perceived waiting time was significantly less than the mean actual waiting time (11.9 min vs 15.5 minutes) for the intervention group (p <0.001); however, the mean perceived waiting time was significantly higher than the mean actual waiting time (32.8 vs 27.03 minutes) for the control group (p < 0.001). The level of satisfaction in the intervention group was significantly higher than that of the control group (p <0.001). There was an inverse relationship between the actual waiting time (Intervention group: r=-0.463; Control group: r= -0.567) and the perceived waiting time (Intervention group: r= -0.439; Control group: r= -0.568) with the satisfaction level in both groups (p < 0.001).

**Conclusion:**

It can be proposed that the application of a queue management system in the emergency department waiting rooms can reduce the actual and perceived waiting times and increase the patient satisfaction.

## 1. Introduction:

Patient satisfaction has transpired as a progressively important issue in health care and is currently used for four associated but different purposes: 1) To measure the quality of care, 2) To evaluate various health care programs or systems, 3) To help organizations notice consumers that are likely to deregister, and 4) To recognize which aspects of a service require improvements to increase patient satisfaction (1). From the early 1990s, research on patients’ satisfaction with emergency care has steadily extended, and many studies have employed a multivariate analytical scheme to point out parameters that mostly anticipate the overall satisfaction in this regard (2). Waiting time has been weighed as a significant determinant of patient satisfaction. Increased waiting time adds to indirect costs of taking part in an emergency department (ED) encounter from the patients’ perspective. In addition, the prolonged waiting time may increase patient disappointment and reduce their sense of control/patience (3). Hence, prognosticators of patient satisfaction in EDs are of great significance. Concentrating on accurate prognosticators may be crucial to sustain current patients and attract potential new ones. Strategic resolutions in this field may also affect the financial suitability of health care institutions (4). 

The movement of patients through a healthcare facility from the point of admission to the point of discharge, which is known as the patient flow, is a critical component of process management in EDs that involves medical care, physical resources, and internal systems, as well as maintaining patient satisfaction and quality of care. Improvement of patient flow in EDs via the application of a queue management system enables efficient handling of resources and patient queues so that patients receive the right care at the right time in a convenient and trouble-free environment. From patient check-in to patient calling and appointment, queue management systems allow the staff to excellently manage patient waiting times and organize the entire patient flow in EDs (2-4). An ED visit frequently yields the ﬁrst and the only impression that patients have with respect to an institution and can markedly shape their post-visit sensations and future reactions (5). Furthermore, a satisﬁed patient departs from the hospital with a positive impression and is less likely to complain or ﬁle suits against the institution (6). In addition to these marketing roots, patient satisfaction has been indicated to increase with discharge instructions. Therefore, physicians can positively influence their patients’ outcomes following the ED visit by warranting the patient satisfaction (7). Queue management system is used to streamline patient flow through hospitals and clinics. Using these systems patients won’t have to wait longer than its necessary, and they can join the que virtually, from anywhere, using SMS, social media, a smartphone app, or a website booking system. 

there are two types of waiting time:

1) The actual time between the patient entering the emergency room and the physician visiting them.

2) The perceived time between the patient entering the emergency room and the physician visiting them.

As service providers, we need to consider both types of waiting time. If we reduce the actual waiting time while enhancing the perceived waiting time, we can improve the patients’ experience. 

Due to the importance of emergency management and patient satisfaction, conditions should be prepared to create a proper and regular queuing system so that patients' waiting time is adjusted and reduced. Therefore, the present study assessed the effect of applying a queue management system on patient satisfaction in ED waiting rooms.

## 2. Methods:

***2.1. Study Design and Setting***

This prospective a single-blinded randomized trial was conducted in Hazrat-e-Ali-Asghar Hospital affiliated to Iran University of Medical Sciences, Tehran, Iran, in 2020 to measure the satisfaction of sick children from their parents’ perspective. The patients’ perception of the waiting time and satisfaction before being visited by an emergency medicine doctor was evaluated with and without applying the queue management system. This study was approved by the Ethics Committee of Iran University of Medical Sciences under the code IR.IUMS.FMD.REC.1398.156. Accordingly, written informed consent was taken from all parents before any intervention. 

***2.2. Participants***

The study sample for this prospective study consisted of all pediatric patients with the Emergency Severity Index of 4 and 5 that presented to the ED during the randomly-selected shifts over 2 months (July-August) in 2020. In all pediatric cases, sick children were the main recipients of health care; however, service providers still required the attention and participation of parents. 

Patients that were randomly selected from the patients that referred to the ED of our hospital. A randomized blocking (block size of four) method was applied using a computer-generated random number list prepared by an investigator with no clinical involvement in the trial.

Eligible patients were randomly divided into two groups of one hundred eighteen using random blocks. This sample size was calculated using the sample size formula for comparing two means. 

 Also, we excluded patients who presented to the ED with severity Index of 1, 2, and 3. Each patient was examined by at least one of the attending emergency physicians, residents, or interns. The staff of the ED of our hospital included attending emergency medicine and pediatric physicians, emergency medicine and pediatric residents, and interns who rotated through the ED for a one-month period.

***2.3. Blinding and Intervention***

This study was single-blinded: the patients and their parents were not aware of the course of the intervention. The researcher and clinicians were informed of the allocation in the intervention group. 

The queue management system employed in the ED of our hospital intervened in the queue length management. The intervention group consisted of patients whose entrance to the triage room until their first visit by a physician was managed using this system. The control group consisted of patients that this system was not used for them but were otherwise under the same conditions as the intervention group. To perform this study, during two months (July-August) in 2020 we randomly selected half of the days and in those days, we used the queue management system. Therefore, patients who came on those specific days were entered to the intervention group.

***2.4. Outcomes and Measurements***

In this study, measuring parents’ satisfaction level was defined as the primary outcome. To measure these values, the actual waiting time and the perceived waiting time measured from the patient’s entrance to the triage room until their visit by a physician were recorded in minutes.

Secondary outcomes were comparing the parent’s stress level, and child’s demographic information and chief complaint between the intervention and control groups. Moreover, patients’ chief complaints were categorized based on the frequency distribution of symptoms including fever, respiratory, gastrointestinal, urinary, and other symptoms.

***2.5. Data Collection***

The data were collected by observation and asking questions; all were gathered in checklists and then recorded in the data bank. Also, parents’ stress and satisfaction levels were also collected using the checklists based on a Likert scale ranging from 1 for very poor to 5 for very good. We didn’t have any missing data in our study.

***2.6. Statistical analysis***

The data were entered into the statistical analysis software, SPSS, version 25, and then statistically analyzed. Kolmogorov-Smirnov test was used to assess the normality of data distribution. All descriptive data had normal distribution. Therefore, the results for the quantitative variables were reported in mean ± standard deviation (SD) format and the ordinal qualitative variables were reported in frequency and percentages. For comparing quantitative and qualitative variables, the Mann-Whitney U, Student’s *t *test or chi-square test were used. *P *values <0.05 were considered statistically significant.

## 3. Results:

Two hundred thirty six patients were assessed (118 patients in each of the control and intervention groups were studied; 58.5% male). The mean age of children in the intervention and control groups was 3.6 ± 2.3 and 3.4 ± 1.8 years, respectively (p = 0.57). Distribution of symptoms was not significantly different between intervention and control groups (p = 0.34; Table 1). There was no significant difference between the two groups in terms of the parents’ stress level on arrival (p = 0.41). 

The mean actual waiting time (15.5 ± 7.5 minutes) as well as the mean perceived waiting time (11.9 ± 7.4 minutes) for the intervention group were significantly lower than those of the control group with the values of 27.03 ± 8.5 and 32.8 ± 8.7 minutes, respectively (p < 0.001). 

The mean perceived waiting time was significantly less than the mean actual waiting time (11.9 min vs 15.5 minutes) for the intervention group (p <0.001); however, the mean perceived waiting time was significantly higher than the mean actual waiting time (32.8 vs 27.03 minutes) for the control group (p < 0.001). 

The level of satisfaction in the intervention group was significantly higher than that of the control group (p <0.001). Moreover, none of the parents reported a very poor, poor, or even average level of satisfaction in the intervention group (all had good and excellent levels of satisfaction; Table 1). 

There was an inverse relationship between the actual waiting time (Intervention group: r=-0.463; Control group: r= -0.567) and the perceived waiting time (Intervention group: r= -0.439; Control group: r= -0.568) with the satisfaction level in both groups (p < 0.001). In other words, the level of satisfaction decreased with increase in the waiting time.

## 4. Discussion:

The findings of this study revealed that application of a queue management system resulted in the decreased perceived waiting time and also the actual waiting time. The presented findings confirm that application of a queue management system could significantly increase the level of satisfaction in the intervention group. An inverse relationship was observed between the actual waiting time and the perceived waiting time with the level of satisfaction. Most of the parents experienced moderate to extreme stress levels on their arrival at the ED.

It is important to consider the demographic properties of the patients, or their parents in the pediatric field, while evaluating the perception of time and their satisfaction (8). 

Franck et al. stated that parental stress was not related to their age, race, or job satisfaction. In contrast, parental stress was independently related to their estimation of pain, the management technique, and the correct information provided by the health care staff about the techniques used for children’s pain reduction and treatment (9). Other studies have also shown that the satisfaction of the patients is not related to their demographic properties or their illness perception. However, the waiting time can affect their satisfaction, significantly (10).

Eight principles can be employed by organizations to affect the customer satisfaction during the waiting time. Based on the presented principles, the waiting time that is not filled with a specific schedule, the waiting time that elapses before offering the treatment, the waiting time accompanied with anxiety, the waiting time for an indefinite period, the waiting time for no apparent reason, the unfair waiting time, and waiting alone seem longer than the actual waiting time. However, the recipient of the service endures a longer waiting time in exchange for receiving a service with a higher value (11).

The study of Spechbach and colleagues in 2019 suggests that there is a golden hour window for patients, which is the time they can wait for receiving medical service. Difference of the triage level with their personal assessment of their condition, having a feeling of being forgotten, ambiguity of the situation, and the lack of sense of privacy were the most important factors, affecting the perceived waiting time in patients in that study (12).

Studies suggest that more than 75%, 68%, and 41% of the patients, regardless of their gender and chief complaint, overestimate their waiting time for triage, after triage, and their total time spent in ED (13). It is shown that young patients and those with less prior ED admission history have more accurate estimations of their waiting time. Patients with longer waiting in the triage also are shown to have overestimations, in comparison with those with longer waiting in the examination room.

Therefore, waiting for an indefinite time in the control group of the present study caused a longer perceived time for this group. Moreover, it seems that applying a queue management system resulted in a more organized atmosphere in the ED waiting room as the actual waiting time was reduced for the intervention group.

Whiting et al. worked on the gap between actual and perceived waiting times and stated that as the gap extends, the satisfaction decreases. Furthermore, as the actual waiting time increases, the waiting gap decreases. Furthermore, although expectations with regard to receiving a service do not affect the waiting gap, they can affect the level of satisfaction. Finally, the higher the applicants’ anxiety, the higher the expectation gap will be (14). The level of satisfaction decreases with increase in the waiting time. Besides, filling the waiting time with fun activities increases the applicants’ satisfaction; however, informing the applicants of the waiting time duration, despite increasing their Perception of waiting time, does not affect their satisfaction (15). The perceived waiting time was, on average, one minute longer than the actual waiting time, and waiting for five minutes or less to receive the service was reasonable for the applicants (15). In contrast, the findings of the current study indicated that the mean perceived waiting time in the intervention group was 3.6 minutes less than the mean actual waiting time, and the mean perceived waiting time was 5.8 more than the mean actual waiting time in the control group. Furthermore, the study conducted by Hui et al. showed that the acceptance of waiting and the manner of response to the waiting time affected applicants’ perception and service evaluation but had no effect on their perceived time. In addition, none of the waiting time-related information pieces applied to the short-term waiting. However, the information related to the waiting time, as compared with the general information, had a greater effect on the average perception of the waiting time in the medium-term waiting time but a lower effect on the average perception of the waiting time in the long-term waiting time (16). The study of Carr and colleagues has revealed that the patients suffer from the ambiguity and uncertainty of their condition, while waiting for the doctor or their surgery (17). Reassuring the patient and his/her company about his condition can be a satisfying factor, decreasing the perceived waiting time.

It should be noted that the perceived time can be influenced by various factors. Even the perceived time for waiting at the red light can be altered with the tempo of the auditory signal, played at the time of the waiting period (18). The condition can be more complicated in a complex situation such as waiting in the ED. Further studies are needed to identify and control these potentially confounding factors. The medical status of the patient, their age, gender, past medical history, and their location in the ED are potential confounders (13, 19). 

Queue management system is especially valuable in developing countries, because the application of the queue management system is not a routine intervention in developing countries (in contrast to developed countries) while it is affordable and suitable for their crowded EDs. 

These types of research-based interventions are required in fields such as clinical care processes, nursing services, and para-clinical services after the ED admission. To observe signs of progress, institutionalization of the quality management in health services is a must, and utilization of the obtained feedback can systematically enhance the efficiency and patient satisfaction in the EDs. Also, we can use this system in the adult emergency department and enhance that place. Therefore, we need to do more research on this topic.

**Table 1:** Comparing the chief complaints, parents' stress, and satisfaction levels between cases handled with (A) and without (B) queue management system

**Variable**	**Group A**		**Group B**		**P**
	**Number**	**Percent**	**Number**	**Percent**	
**Chief complaints**					
Fever	47	39.8	51	43.2	0.34
Respiratory	2	1.7	7	5.9	
Gastrointestinal	29	24.6	25	21.2	
Urinary	4	3.4	6	5.1	
Others	36	30.5	29	24.6	
**Stress level**					
No stress	0	0	0	0	0.41
Low	0	0	4	3.4	
Moderate	28	23.7	26	22	
High	48	40.7	52	44.1	
Extreme	42	35.6	36	30.5	
**Satisfaction level**					
Very poor	0	0	1	0.8	< 0.001
Poor	0	0	11	9.3	
Average	0	0	54	45.8	
Good	16	13.6	49	41.5	
Excellent	102	86.4	3	2.5	

## 5. Limitations

This research was subject to several limitations. The first limitation was related to its conduction in a single center with a small number of participants. The second limitation was related to the single-blinded nature of the study. Moreover, although the researchers tried to control the effect of other aspects of this study on the data, it was possible that applying a queue management system affected the triage nurses’ behavior toward the patients’ family. We had some biases and confounding conditions such as lack of time to accurately enter data to checklist or the fatigue of physicians and interns while visiting and talking to the patients. Also, how the ED staff talks and behave in front of parents affects the answers to questions on the checklists.

## 6. Conclusion:

The findings of this study revealed that with regards to providing the optimal ED services and gaining patients’ satisfaction, the application of a queue management system can result in a remarkable change in the health care system as it affects the actual and perceived waiting times. 

## 7. Declarations

### 7.1. Consent for publication

Not applicable. 

### 7.2. Availability of data and materials

The datasets used and analyzed in the current study are available from the corresponding author on reasonable request.

### 7.3. Competing interest

None declared.

### 7.4. Funding

None declared.

### 7.5. Author contributions

Study concept and design, acquisition of the data: Ali Bidari, Shabahang Jafarnejad

Analysis and interpretation of the data: Shabahang Jafarnejad, Nazanin Alaei Faradonbeh 

Drafting of the manuscript: Nazanin Alaei Faradonbeh

Critical revision of the manuscript for important intellectual content: Ali Bidari,

Statistical expertise: Ali Bidari, Nazanin Alaei Faradonbeh

Acquisition of funding: None.

### 7.6. Acknowledgments

The authors would like to express their deepest appreciation to the Hazrat-e-Ali-Asghar pediatric hospital emergency department’s staff for their valuable cooperation.
